# Effect of laser treatment on postural control parameters in patients with chronic nonspecific low back pain: a randomized placebo-controlled trial

**DOI:** 10.1590/1414-431X20198474

**Published:** 2019-11-21

**Authors:** J. Taradaj, K. Rajfur, J. Rajfur, K. Ptaszkowski, L. Ptaszkowska, M. Sopel, J. Rosińczuk, R. Dymarek

**Affiliations:** 1 Institute of Physiotherapy and Health Sciences, Academy of Physical Education, Katowice, Poland; 2 College of Rehabilitation Sciences, University of Manitoba, Winnipeg, Canada; 3 Faculty of Physiotherapy, Opole Medical School, Opole, Poland; 4 Department of Physiotherapy, Wroclaw Medical University, Wroclaw, Poland; 5 Department of Nervous System Diseases, Wroclaw Medical University, Wroclaw, Poland

**Keywords:** Laser therapy, Low back pain, Physical therapy modalities, Postural balance

## Abstract

The management of nonspecific lumbar pain (NSLP) using laser irradiation remains controversial. A systematic review of recently published studies indicates that the effects of laser therapy are commonly assessed using only imperfect methods in terms of measurement error. The main objective of this study was to assess static postural stability using an objective tool in patients with chronic NSLP after laser irradiation at different doses and wavelengths. In total, 68 patients were included in the laser sessions and were randomly assigned into four groups: high-intensity laser therapy at 1064 nm and 60 J/cm^2^ for 10 min (HILT), sham (HILT placebo), low-level laser therapy at 785 nm and 8 J/cm^2^ for 8 min (LLLT), and sham (LLLT placebo). In addition, all patients were supplemented with physical exercises (standard stabilization training). To assess postural stability, a double-plate stabilometric platform was used. All measurements were performed pre- and post-laser sessions (three weeks) and at follow-up time points (one and three months). Laser procedures led to more balanced posture stability in patients, although these positive changes were significant mainly for short-term observation (after 4-week therapy). In the follow-up analysis, the parameters were gradually impaired. Kruskal-Wallis analysis of variance (ANOVA) for independent variables did not show any difference between the studied groups. Low- and high-intensity laser therapy does not lead to a significant improvement in postural sway in patients with NSLP compared with standard stabilization training based on short- and long-term observations.

## Introduction

Laser irradiation in the management of nonspecific lumbar pain (NSLP) remains an area of much confusion and controversy. Numerous scientific reports in the literature demonstrate the significant utility and clinical efficacy of laser therapy (1–4). Nevertheless, it should be noted that other more critical studies testify to the lack of purposefulness of laser radiation (5,6).

The goal of laser irradiations in NLSP is a change in normal life, e.g., motor and body balance control, recovery of normal postural sway, and complete physical fitness. Laser therapy offers a specific dose of energy (photons) to the areas of the tissue to be treated. Laser light falling on the surface of the patient's skin and subsequently on the border between successive structures, such as subcutaneous tissue, muscles, and ligaments, is subject to the laws of physics. However, despite the occurrence of wave reflection, refraction, and scattering, laser light is able to penetrate the hernia of the spinal disc and periarticular structures (7,8).

Researchers report that the effect of laser radiation at the cellular level is manifested by increased production of ATP, increased activity of membrane enzymes, increased synthesis of DNA and RNA, and acceleration of electrolyte exchange between the cell and the surrounding areas (7,9). At the tissue level, acceleration of blood and lymph circulation, reduced intracapillary pressure, increased excitability threshold of nerve endings, and stimulation of immune response are observed.

The phenomena described above constitute the basis for the described analgesic and anti-inflammatory mechanisms. It is believed that laser therapy suppresses the release of inflammatory mediators, reduces edema, and increases activation of descending anti-nociceptive system and hyperpolarization of primary nerve endings. In conclusion, the observed remission of pain and inflammatory symptoms should correspond with improvement in the patient's functional status (8,10,11).

Recent data demonstrate that it is also impossible to exclude the fact that researchers generally evaluate the effectiveness of laser therapy based only on imperfect tests (e.g., Lasàgue's or Schober's) and questionnaires in terms of measurement error. In addition, the questionnaires used analyze only subjective (i.e., indicated by the patient) pain sensations as well as functional abilities and disturbances in mobility (1–16,12–14).

The authors of this study do not question the need for using simple scales, surveys, and questionnaires in scientific research. Undoubtedly, the tools mentioned above play an important role in clinical practice and allow relatively easy verification of the results obtained by other physiotherapists in their work in a hospital, clinic, or private practice. However, the principles and recommendations of evidence-based medicine should be used to unambiguously verify the utility of laser therapy in musculoskeletal disorders. For this purpose, modern tools should also be used in physiotherapy research, demonstrating significant repeatability of measurements and objectivity of results.

Computerized posturography testing is a valuable and objective technique for measuring postural strategies under challenging static and dynamic conditions (15). The strategy allows assessment of balance control with highly repeatable measurements, which determines the efficiency of the balance and proprioception systems, especially the coordination functions of the nervous and muscular systems (16). Proprioception participates in the maintenance of a standing posture of the body by constantly regulating center of pressure (COP) movements necessary for orthogonal projection of the center of gravity (COG) during the postural phase (17). Posturography is an important diagnostic tool for evaluating balance disorders and developing an individual rehabilitation program for each patient to control his/her progress (18).

Patients with NSLP show proprioceptive deficits of the trunk concerning the anteroposterior axis, which affect the balance of posture (19). The posturography device captures the movement of the COG and simultaneously calculates the point of application of the resultant ground reaction force known as the COP. Observations of the COP and asymmetry in the burden placed on feet in patients with NSLP using objective tools, such as a stabilometric (posturographic) test, may play an essential role in monitoring postural balance performance and the ongoing management progression. This technique also allows for fully conscious control of the complex treatment process and offers the possibility to verify hypotheses of scientific studies (20,21).

According to the information presented above, the main goal of this paper was to measure static postural stability using precise and objective tools among patients with NSLP after laser irradiations at different doses and wavelengths based on the analysis of short- and long-term results with comparison to the placebo effect. The authors of this study hypothesized that laser irradiations would improve postural control of patients with NSLP compared to placebo interventions in both short- and long-term observations. It was assumed that effects described in the literature (6–9,12,13) should positively correspond with improvement in the patient's functional status. It was also assumed that high-intensity laser therapy (HILT) would present much more explicit and permanent effect for postural sway.

## Material and Methods

### Study design

The present study was a randomized controlled clinical trial conducted between February 2016 and March 2017 at the Laboratory of Functional Tests at the Faculty of Physiotherapy of Public Higher Medical Professional School in Opole, Poland and the College of Rehabilitation Sciences in Manitoba, Canada.

### Qualification

The qualification of patients was assessed by a team including an orthopedist, a neurologist, a neurosurgeon, an internist, a radiologist, and a physical therapist. Patients with diagnosed lumbar hernia disc and nonspecific chronic pain syndrome with symptom peripheralization into the lower extremity without neurological deficit and history of previous surgery of the spine were included in the trial. The NSLP diagnosis was based on magnetic resonance imaging examination that determined the advancement of degenerative and inflammatory changes of the lumbar region (>Modic III).

### Exclusion criteria

The exclusion criteria were as follows: 1) acute and subacute pain episodes in the lumbar region; 2) sciatica episodes; 3) degenerative changes of cervical or thoracic region; 4) past fractures of the bone structures of the spine; 5) vertebral column tumors, intradural and intramedullary tumors; 6) vertebra forward dislocation; 7) rheumatoid arthritis and ankylosing spondylitis; 8) cauda equina syndrome; 9) pregnancy or ovulation; 10) acute and chronic cardiovascular diseases; 11) arrhythmia and implanted pacemaker; 12) implanted metal implants; 13) dermatological conditions in the area of irradiation; 14) sensory deficits; 15) psychiatric disorders; 16) immunological diseases; 17) infections and elevated temperature; 18) chronic drug use; 19) problems with the balance system, labyrinth, and inner ear; and 20) other central nervous system diseases.

### Participants

**Figure 1.**
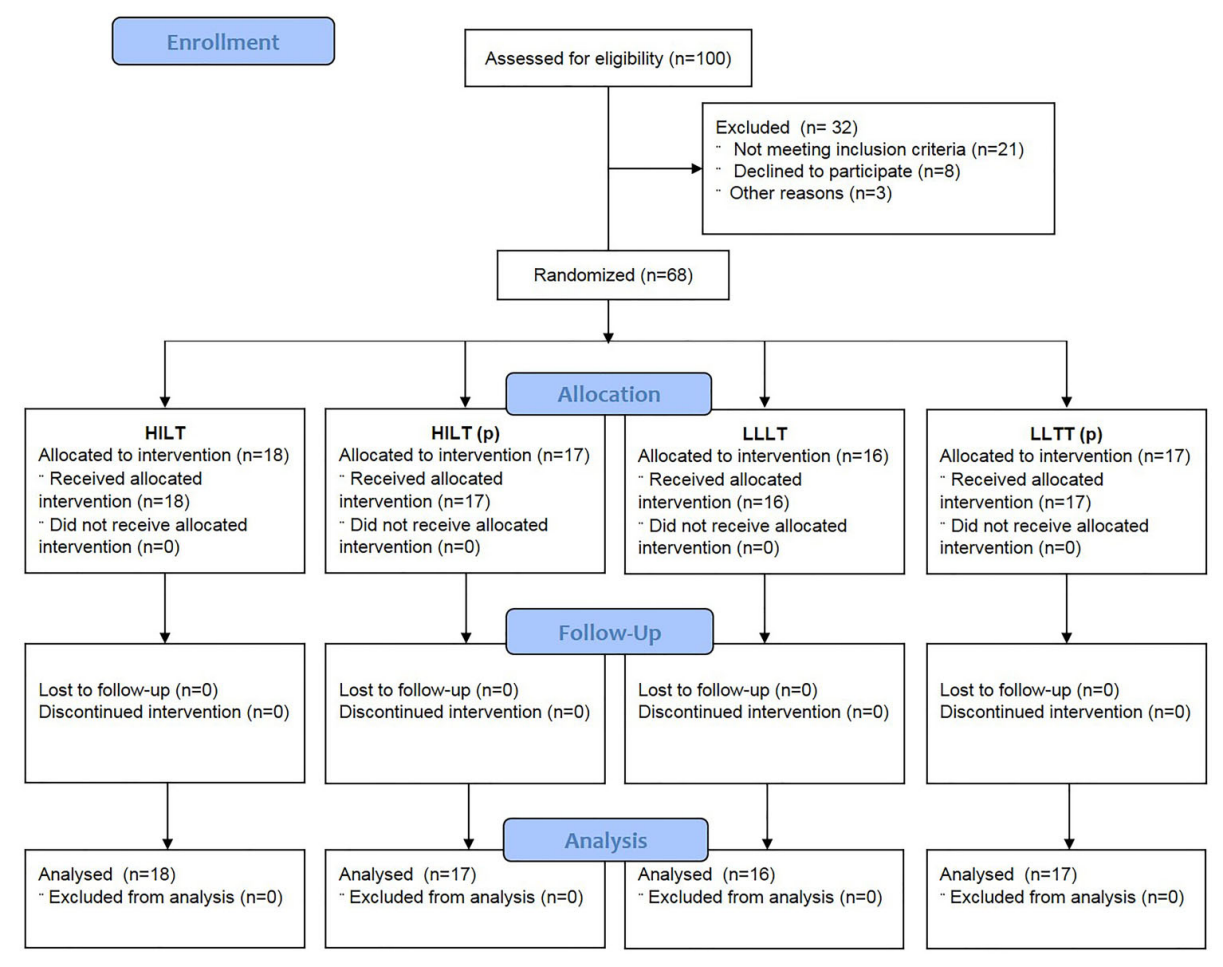
CONSORT flowchart of patients' recruitment and study flow. HILT: high-intensity laser therapy group; HILT (p): high-intensity laser therapy sham group; LLLT: low-level laser therapy group; LLLT (p): low-level laser therapy sham group.

Finally, 68 patients were randomly assigned using a computer number generator into four groups to receive laser therapy sessions. All groups were similar with no differences in baseline characteristics regarding demographic and other factors, such as pain level, functional condition, and range of motion in joints. A thorough analysis of the homogeneity of patients is presented in Table 1. A detailed flow of participants at each stage of the project based on CONSORT (Consolidated Standards of Reporting Trials) guidelines is shown in Figure 1.

**Table 1.** Demographic characteristics of participants in the study.

Character	Group	N	Mean	Median	Min	Max	Q25	Q75	SD	P value
Age (years)	HILT	18	44.67	44.00	29.00	58.00	41.00	48.00	4.96	0.8436
	HILT (p)	17	44.24	45.00	26.00	51.00	41.00	47.00	4.34	
	LLLT	16	45.19	45.50	29.00	53.00	42.00	47.50	4.17	
	LLLT (p)	17	45.76	52.00	22.00	76.00	36.00	56.00	15.04	
Height (cm)	HILT	18	168.7	169.5	162.0	175.0	164.0	172.0	4.26	0.7176
	HILT (p)	17	169.4	172.0	158.0	181.0	159.0	175.0	7.98	
	LLLT	16	168.9	168.0	156.0	176.0	168.0	172.0	4.57	
	LLLT (p)	17	169.8	170.0	164.0	177.0	168.0	171.0	2.96	
Body weight (kg)	HILT	18	74.17	75.00	57.00	90.00	65.00	83.00	11.41	0.9674
	HILT (p)	17	73.94	75.00	54.00	92.00	65.00	84.00	11.81	
	LLLT	16	75.38	75.00	59.00	92.00	62.00	90.00	12.99	
	LLLT (p)	17	76.06	78.00	55.00	87.00	74.00	82.00	8.89	
BMI (kg/m2)	HILT	18	25.96	26.64	21.19	31.14	22.76	28.39	3.11	0.9782
	HILT (p)	17	25.69	25.35	21.36	30.46	22.58	28.44	3.25	
	LLLT	16	26.42	26.25	19.05	31.89	21.97	30.42	4.31	
	LLLT (p)	17	26.36	27.04	18.59	28.38	26.93	27.77	2.86	
Duration of disease (months)	HILT	18	55.89	57.00	46.00	64.00	51.00	60.00	5.97	0.9610
	HILT (p)	17	55.41	56.00	46.00	64.00	52.00	60.00	5.96	
	LLLT	16	54.56	56.00	36.00	68.00	48.50	61.50	8.88	
	LLLT (p)	17	56.47	58.00	47.00	65.00	52.00	60.00	5.68	
Pain (VAS)	HILT	18	7.22	8.00	4.00	10.00	5.00	9.00	1.96	0.0931
	HILT (p)	17	7.59	8.00	5.00	9.00	7.00	9.00	1.42	
	LLLT	16	8.50	9.00	5.00	10.00	8.00	9.50	1.55	
	LLLT (p)	17	7.18	7.00	5.00	10.00	6.00	8.00	1.67	
Pain (LATQ)	HILT	18	9.22	8.00	5.00	15.00	7.00	11.00	3.08	0.1047
	HILT (p)	17	8.65	9.00	4.00	13.00	6.00	12.00	3.24	
	LLLT	16	7.75	8.00	4.00	12.00	6.00	9.00	2.32	
	LLLT (p)	17	6.94	6.00	4.00	12.00	5.00	9.00	2.66	
Schober test (cm)	HILT	18	3.00	3.00	2.00	4.00	3.00	3.00	0.59	0.2889
	HILT (p)	17	2.71	2.50	2.00	4.00	2.00	3.00	0.64	
	LLLT	16	3.19	3.00	2.00	4.00	3.00	3.50	0.54	
	LLLT (p)	17	4.12	4.00	3.00	5.00	3.00	5.00	0.86	
Laseque test - left extremity (^o^)	HILT	18	60.83	60.00	25.00	85.00	55.00	75.00	17.84	0.2467
	HILT (p)	17	39.71	35.00	25.00	60.00	30.00	50.00	11.38	
	LLLT	16	54.69	57.50	30.00	80.00	30.00	70.00	18.84	
	LLLT (p)	17	56.18	55.00	35.00	75.00	50.00	65.00	13.17	
Laseque test - right extremity (^o^)	HILT	18	61.67	60.00	40.00	80.00	55.00	70.00	12.72	0.2045
	HILT (p)	17	38.24	30.00	30.00	60.00	30.00	45.00	12.11	
	LLLT	16	52.81	57.50	25.00	75.00	30.00	70.00	18.71	
	LLLT (p)	17	58.53	65.00	30.00	75.00	50.00	65.00	13.32	

Min: minimum value; Max: maximum value; Q25: lower quartile; Q75: upper quartile; SD: standard deviation; HILT: high-intensity laser therapy group; HILT (p): high-intensity laser therapy sham group; LLLT: low-level laser therapy group; LLLT (p): low-level laser therapy sham group. BMI: body mass index; VAS: visual analog scale; LATQ: Laitinen questionnaire. Statistical analysis was done with ANOVA.

All participants signed a written consent form before participating in the study, which was approved by the Bioethical Commission of the Medical University of Wroclaw, Poland (No. KB–666/2015).

### Interventions

**Figure 2.**
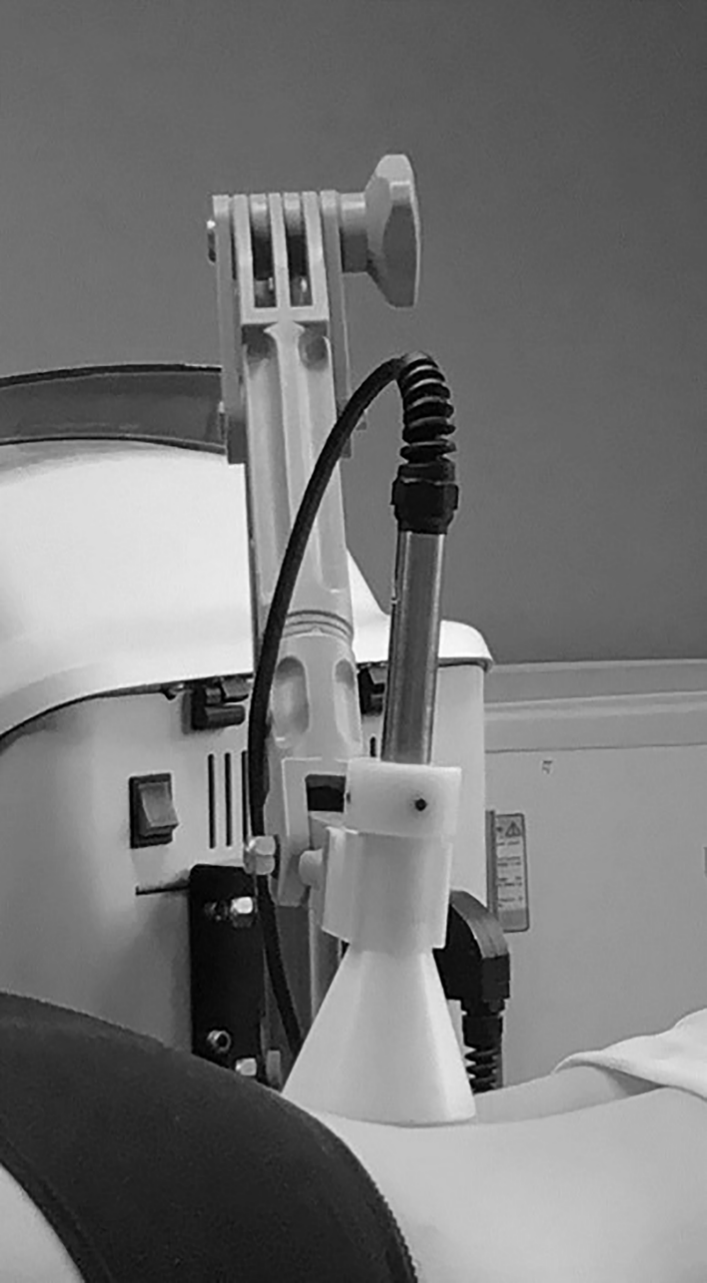
Patient during the high-intensity laser therapy application.

The high-intensity laser therapy (HILT) group (n=18; 10 male patients, 8 female patients) received high-intensity laser irradiation with constant wave, contact method, stable technique, spot applicator with a 30 cm^2^ applicator above a lumbar area of 6×5 cm (Figure 2), wavelength (λ) of 1064 nm, energy (E) of 60 J/cm^2^, and duration (d) of 10 min. The placebo (HILT-p) group (n=17; 9 male patients, 8 female patients) underwent placebo irradiations using a passive HILT procedure (10 min of a single application).

**Figure 3.**
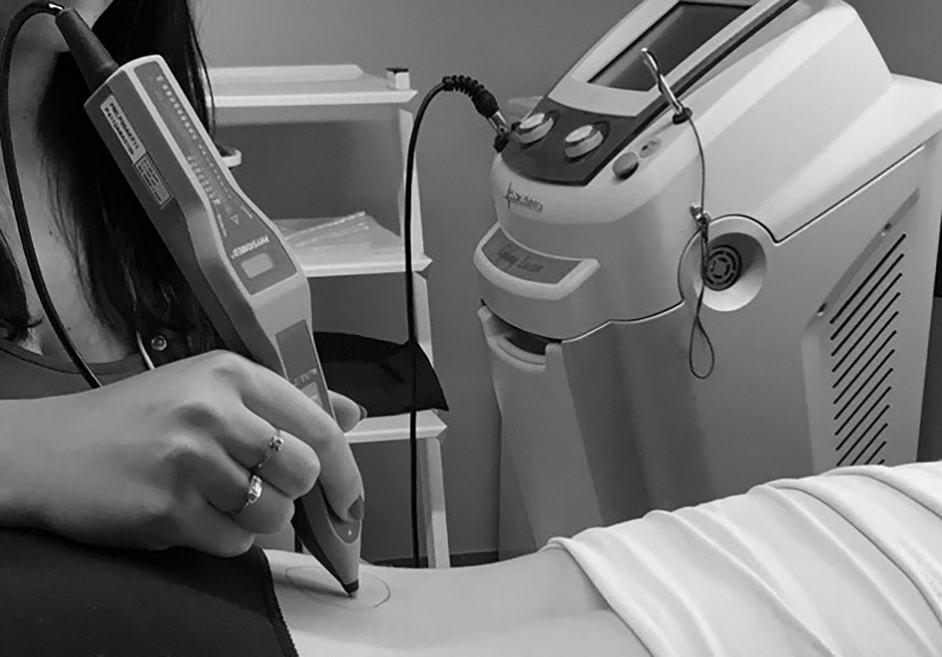
Patient during the low-level laser therapy application.

The low-level laser therapy (LLLT) group (n=16; 8 male patients, 8 female patients) was exposed to low-energy laser irradiation with constant wave, contact method, stable technique, spot applicator in the lower back paraspinal region, λ=785 nm, E=8 J/cm^2^, and d=8 min (Figure 3). The placebo (LLLT-p) group (n=17; 9 male patients, 8 female patients) received a series of placebo irradiations with a passive LLLT procedure (8 min of a single application).

In this study, the single-blind method (patients were blinded) was used where the laser device generated a visible red light, but the treatment parameters were zeroed (the device was turned off). Sessions of 15 irradiations conducted every day five times a week for three weeks were delivered to the patients from all groups. The Cyborg Laser apparatus (Cosmogamma, Indonesia) with a Gallium-Aluminum-Arsenide (Ga-Al-As) laser diode was used in the HILT and HILT-p groups. In contrast, the LAS-Expert apparatus (Physiomed Electromedizin, Germany) with a He-Ne laser diode was used in the LLLT and LLLT-p groups.

Additionally, laser irradiation in all patients was supplemented with physical exercises performed throughout the therapy period. A single series lasted 45 min and was performed five times per week (Monday to Friday). Stabilization training included the following: 1) techniques for the relaxation of the myofascial system on erector spinae muscle; 2) techniques for activating the neutral position of the lumbopelvic complex and deep muscles; 3) stimulation of proper breathing and correct activation of the transverse abdominal muscle; 4) coordination of superficial and deep muscles activation; and 5) postural and dynamic training.

### Measurements

An objective measurement tool for evaluating postural stability under static body conditions was used in this research. For this purpose, the assessment was performed using a double-plate stabilometric platform compatible with a computer-aided posturographic system model CQ Stab 2P (CQ Electronic System, Poland) with an assumed measurement error of 0.86%.

The CQ Stab platform makes it possible to perform the analysis of the measured parameters for each leg separately. We have chosen the option of measurement of both legs simultaneously with the control of loading the platform through the right leg (50%) *vs* left (50%) on the computer monitor. The platform is equipped with a system of tensometric sensors responsible for registering changes in the COP and its trajectory during stance, which determines the individual postural control system (as it is indicative of the stability of the system).

**Figure 4.**
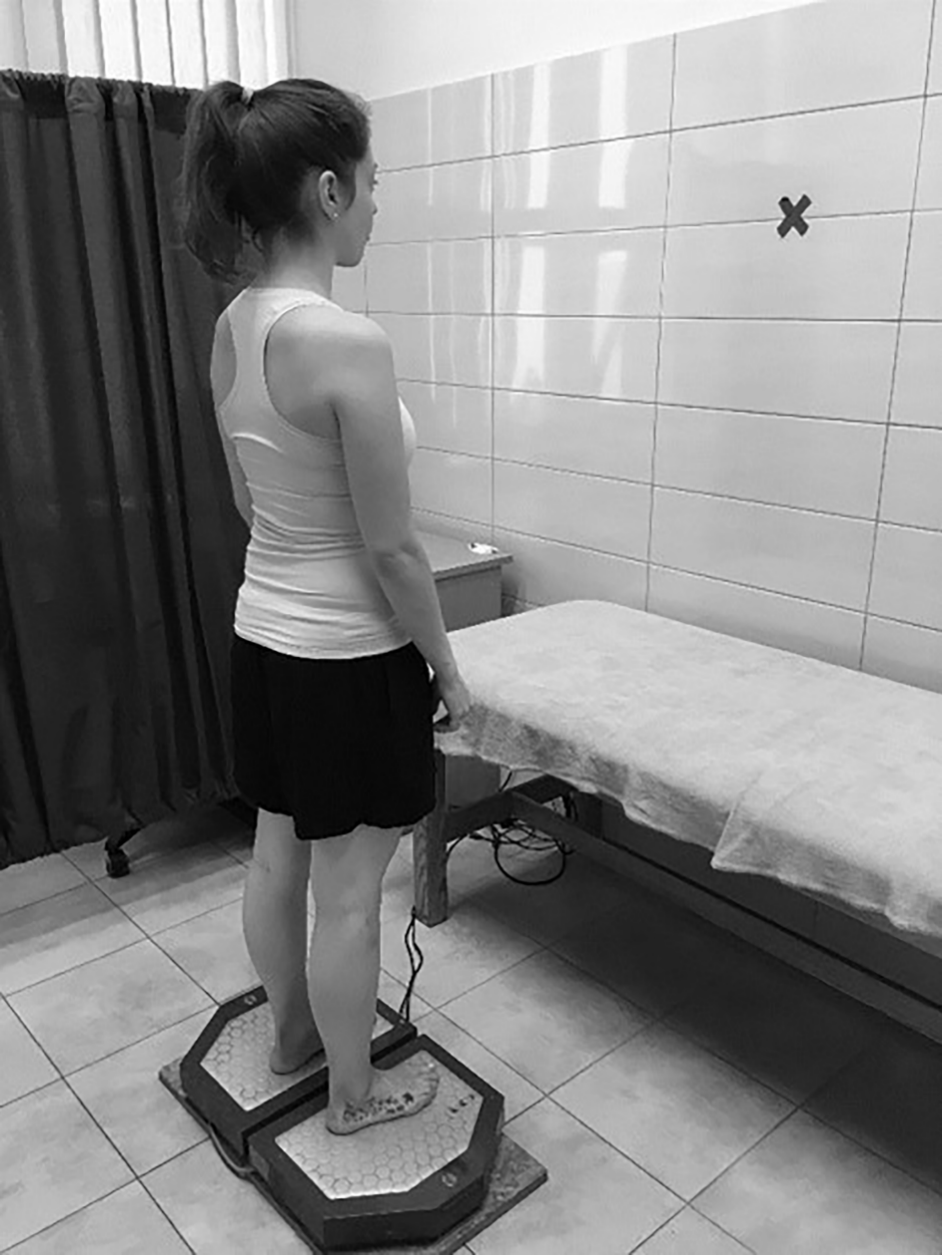
Patient during the static postural stability measurement.

Two 60-s quiet standing trials with arms relaxed by the sides were conducted with the eyes open (30 s) looking straight ahead at a wall 1.5 m away and with the eyes closed (30 s). After each trial, subjects stepped off the platform and rested up to 1 min to avoid any discomfort (Figure 4). COP signals transmitted from the force plate were amplified and sampled at the frequency of 100 samples per second. Signals were filtered at a 7-Hz cut-off frequency.

The signals from the force sensors were amplified in initial measuring amplifier and then processed in an analog-to-digital converter to digital form for transfer to the control and communication module. Module communication control was responsible for the process of collecting data from A/D converters and sending measurement data to a PC. Software installed on the PC was responsible for converting the measurement results. Analysis, display, and printing tests were performed using the software. All patient data and images were saved in the computer's memory and may be easily transferred to a pen drive or CD.

To assess the postural stability of patients, the most commonly used parameters were analyzed: 1) sway path, which is the total distance of the COP displacement during the test (mm); 2) sway path along the Y-axis, which is the anteroposterior path length in the sagittal plane (mm); 3) sway path along the X-axis, which is the medio-lateral path length in the frontal plane (mm); 4) mean velocity, which is the COP sway displacement in all planes (mm/s); 5) mean sway frequency, which is the frequency of COP sway displacement in all planes (Hz); and 6) the sway area, which is the total sway surface enclosed by the path of COP during the test (mm^2^). According to the literature, it was assumed that the lower the value of the above parameters, the better the control of postural stability.

All assessments were conducted before and after a series of laser irradiations. Then, after one and three months following the last laser session, analogical assessments were repeated to determine the long-term result (follow-up). During this period, the patients did not participate in any therapies that could affect the results. Additionally, it should be emphasized that all procedures with laser irradiation were performed by the same physiotherapist. Similarly, all control-diagnostic measurements were performed by the same lab technician.

### Statistical analysis

Results were analyzed using STATISTICA 12 software (StatSoft Inc., USA). For arithmetic variables, arithmetic means, standard deviations, medians, range of variation (extreme values), and quartiles were calculated. The Shapiro-Wilk test was used to determine the type of distribution for all quantitative variables. Intergroup comparisons were calculated using the nonparametric Kruskal-Wallis ANOVA test with multiple comparisons. Intragroup comparisons were calculated using the Friedman ANOVA test with multiple comparisons. Here, P≤0.05 was considered statistically significant. Based on type I error, a probability of 0.05, and 90% power, the detection of statistically significant differences between four groups required at least 15 patients in each group (total of 60 patients).

## Results

After completing the study, beneficial effects on postural stability parameters were observed in all patients in the tests with open and closed eyes (Tables 2 to [Table t03] [Table t04] [Table t05] [Table t06] 7). Only the mean sway frequency changes were not statistically significant (Table 6). Procedures undertaken in the groups led to improved posture stability in the patients; however, these positive changes were significant mainly in short-term observations (after 3-week therapy). Unfortunately, it was also noted that in the follow-up analysis (1 and 3 months after therapy without continuing stabilization exercises), the parameters were gradually impaired. This finding indicated that all these positive changes in the results were very unstable and only observed for a short-term period.

**Table 2.** Sway path results (mm).

	Before therapy mean (SD)	After therapy mean (SD)	1-month follow-up mean (SD)	3-months follow-up mean (SD)	^*^P value
Open eyes					
HILT	197.83 (35.49)	123.44 (42.22)	156.06 (31.78)	193.83 (42.60)	0.0281
HILT (p)	202.56 (42.89)	130.07 (50.33)	163.78 (42.31)	196.68 (50.12)	0.0220
LLLT	193.77 (34.88)	128.44 (41.03)	157.01 (45.78)	185.77 (48.90)	0.0288
LLLT (p)	198.49 (31.33)	125.56 (40.12)	160.11 (40.21)	193.70 (51.34)	0.0250
^**^P value	0.4550	0.5455	0.4980	0.5380	
Closed eyes					
HILT	282.50 (85.03)	206.62 (68.92)	259.67 (73.73)	274.44 (114.70)	0.0310
HILT (p)	275.34 (90.21)	198.45 (70.27)	256.89 (80.12)	272.89 (120.78)	0.0278
LLLT	281.56 (84.32)	202.89 (65.89)	260.01 (70.27)	274.21 (103.72)	0.0312
LLLT (p)	279.21 (83.88)	200.78 (67.90)	262.80 (80.11)	280. 11 (130.34)	0.0316
^**^P value	0.4670	0.4878	0.5110	0.5122	

*Friedman ANOVA, level of significance (before *vs* after *vs* 1-month follow-up *vs* 3-months follow-up). **Kruskal-Wallis ANOVA, level of significance (HILT *vs* HILT (p) *vs* LLLT *vs* LLLT (p) group). SD: standard deviation; HILT: high-intensity laser therapy group; HILT (p): high-intensity laser therapy sham group; LLLT: low-level laser therapy group; LLLT (p): low-level laser therapy sham group.

**Table 3. t03:** Sway path along the Y-axis results (mm).

	Before therapy mean (SD)	After therapy mean (SD)	1-month follow-up mean (SD)	3-months follow-up mean (SD)	^*^P value
Open eyes					
HILT	140.56 (27.09)	109.33 (36.39)	116.65 (42.11)	133.02 (43.08)	0.0410
HILT (p)	142.01 (23.34)	112.76 (39.89)	120.80 (43.29)	140.08 (45.88)	0.0422
LLLT	146.03 (27.83)	115.01 (38.33)	122.02 (40.22)	140.11 (50.01)	0.0422
LLLT (p)	139.44 (20.86)	108.99 (38.45)	115.88 (40.71)	135.02 (50.44)	0.0420
^**^P value	0.5788	0.2880	0.4060	0.5010	
Closed eyes					
HILT	232.61 (84.43)	189.77 (89.67)	197.22 (92.01)	227.08 (92.76)	0.0466
HILT (p)	235.11 (84.22)	192.18 (90.78)	199.03 (92.11)	228.06 (93.89)	0.0468
LLLT	238.02 (90.11)	195.07 (92.06)	201.45 (92.77)	227.99 (98.05)	0.0466
LLLT (p)	231.10 (80.12)	183.39 (80.78)	194.02 (90.70)	226.20 (100.04)	0.0476
^**^P value	0.7677	0.4180	0.5166	0.7890	

*Friedman ANOVA, level of significance (before *vs* after *vs* 1-month follow-up *vs* 3-months follow-up). **Kruskal-Wallis ANOVA, level of significance (HILT *vs* HILT (p) *vs* LLLT *vs* LLLT (p) group). SD: standard deviation; HILT: high-intensity laser therapy group; HILT (p): high-intensity laser therapy sham group; LLLT: low-level laser therapy group; LLLT (p): low-level laser therapy sham group.

**Table 4. t04:** Sway path along the X-axis results (mm).

	Before therapy mean (SD)	After therapy mean (SD)	1-month follow-up mean (SD)	3-months follow-up mean (SD)	^*^P value
Open eyes					
HILT	100.01 (22.19)	79.07 (23.33)	86.55 (24.02)	97.99 (24.88)	0.0355
HILT (p)	99.12 (20.02)	75.03 (22.88)	84.88 (25.02)	93.89 (25.12)	0.0300
LLLT	101.06 (21.86)	78.67 (23.04)	87.01 (24.76)	99.10 (25.25)	0.0350
LLLT (p)	99.88 (20.13)	78.77 (22.03)	88.80 (23.11)	97.22 (24.33)	0.0350
^**^P value	0.7710	0.6890	0.6678	0.7010	
Closed eyes					
HILT	116.22 (24.10)	90.57 (24.33)	100.01 (24.37)	111.23 (25.06)	0.0450
HILT (p)	112.56 (22.11)	89.08 (23.04)	100.44 (23.21)	108.02 (24.88)	0.0422
LLLT	119.66 (20.10)	93.30 (22.02)	94.99 (22.79)	107.03 (24.05)	0.0447
LLLT (p)	112.19 (21.78)	90.80 (23.01)	97.89 (23.01)	110.03 (23.05)	0.0451
^**^P value	0.7102	0.7450	0.4569	0.6200	

*Friedman ANOVA, level of significance (before *vs* after *vs* 1-month follow-up *vs* 3-months follow-up). **Kruskal-Wallis ANOVA, level of significance (HILT *vs* HILT (p) *vs* LLLT *vs* LLLT (p) group). SD: standard deviation; HILT: high-intensity laser therapy group; HILT (p): high-intensity laser therapy sham group; LLLT: low-level laser therapy group; LLLT (p): low-level laser therapy sham group.

**Table 5. t05:** Mean velocity results (mm/s).

	Before therapy mean (SD)	After therapy mean (SD)	1-month follow-up mean (SD)	3-months follow-up mean (SD)	^*^P value
Open eyes					
HILT	6.58 (1.18)	4.02 (1.22)	5.12 (1.24)	5.97 (1.43)	0.0368
HILT (p)	6.50 (1.23)	4.01 (1.23)	5.03 (1.20)	5.89 (1.25)	0.0370
LLLT	6.62 (1.37)	4.19 (1.35)	5.21 (1.34)	6.01 (1.45)	0.0402
LLLT (p)	6.64 (1.50)	4.16 (1.32)	5.16 (1.34)	5.92 (1.34)	0.0370
^**^P value	0.5780	0.4889	0.4880	0.5330	
Closed eyes					
HILT	9.42 (2.83)	6.06 (3.01)	7.56 (3.11)	8.04 (3.55)	0.0318
HILT (p)	9.31 (2.08)	5.89 (3.12)	7.50 (3.30)	8.34 (3.67)	0.0318
LLLT	10.02 (3.11)	6.57 (4.11)	7.74 (3.12)	8.40 (3.44)	0.0278
LLLT (p)	10.05 (3.21)	6.70 (4.12)	7.82 (4.30)	8.42 (3.67)	0.0276
^**^P value	0.1138	0.1160	0.6110	0.7205	

*Friedman ANOVA, level of significance (before *vs* after *vs* 1-month follow-up *vs* 3-months follow-up). **Kruskal-Wallis ANOVA, level of significance (HILT *vs* HILT (p) *vs* LLLT *vs* LLLT (p) group). SD: standard deviation; HILT: high-intensity laser therapy group; HILT (p): high-intensity laser therapy sham group; LLLT: low-level laser therapy group; LLLT (p): low-level laser therapy sham group.

**Table 6. t06:** Mean frequency results (Hz).

	Before therapy mean (SD)	After therapy mean (SD)	1-month follow-up mean (SD)	3-months follow-up mean (SD)	^*^P value
Open eyes					
HILT	0.39 (0.18)	0.36 (0.22)	0.36 (0.23)	0.37 (0.23)	0.8618
HILT (p)	0.35 (0.21)	0.35 (0.24)	0.36 (0.23)	0.36 (0.25)	0.9022
LLLT	0.36 (0.21)	0.35 (0.25)	0.36 (0.25)	0.37 (0.25)	0.9112
LLLT (p)	0.36 (0.18)	0.34 (0.20)	0.37 (0.22)	0.36 (0.23)	0.9170
^**^P value	0.7700	0.7890	0.7820	0.7330	
Closed eyes					
HILT	0.48 (0.20)	0.45 (0.25)	0.48 (0.31)	0.50 (0.31)	0.7180
HILT (p)	0.51 (0.24)	0.47 (0.30)	0.47 (0.30)	0.49 (0.31)	0.7060
LLLT	0.51 (0.25)	0.48 (0.30)	0.49 (0.29)	0.50 (0.30)	0.7205
LLLT (p)	0.50 (0.21)	0.48 (0.31)	0.50 (0.30)	0.51 (0.32)	0.7200
^**^P value	0.6890	0.6678	0.6780	0.7005	

*Friedman ANOVA, level of significance (before *vs* after *vs* 1-month follow-up *vs* 3-months follow-up). **Kruskal-Wallis ANOVA, level of significance (HILT *vs* HILT (p) *vs* LLLT *vs* LLLT (p) group). SD: standard deviation; HILT: high-intensity laser therapy group; HILT (p): high-intensity laser therapy sham group; LLLT: low-level laser therapy group; LLLT (p): low-level laser therapy sham group.

**Table 7.** Sway area results (mm^2^).

	Before therapy mean (SD)	After therapy mean (SD)	1-month follow-up mean (SD)	3-months follow-up mean (SD)	^*^P value
Open eyes					
HILT	180.50 (87.73)	110.21 (70.21)	130.93 (72.03)	171.32 (73.10)	0.0338
HILT (p)	178.11 (86.32)	108.08 (69.44)	127.04 (70.22)	170.30 (71.31)	0.0350
LLLT	178.59 (89.21)	109.11 (70.25)	125.45 (72.25)	165.09 (73.05)	0.0352
LLLT (p)	179.28 (86.88)	109.02 (67.89)	127.08 (70.04)	168.47 (70.99)	0.0289
^**^P value	0.7702	0.8228	0.6112	0.6102	
Closed eyes					
HILT	374.44 (213.15)	312.62 (215.11)	355.12 (215.24)	362.08 (216.05)	0.0387
HILT (p)	370.55 (210.12)	310.72 (211.32)	349.29 (213.13)	359.88 (214.62)	0.0390
LLLT	368.88 (207.03)	309.66 (210.13)	351.26 (210.49)	360.36 (212.03)	0.0392
LLLT (p)	371.03 (210.12)	310.22 (210.89)	349.98 (211.23)	361.01 (112.43)	0.0380
^**^P value	0.8442	0.8878	0.8889	0.8980	

*Friedman ANOVA, level of significance (before *vs* after *vs* 1-month follow-up *vs* 3-months follow-up). **Kruskal-Wallis ANOVA, level of significance (HILT *vs* HILT (p) *vs* LLLT *vs* LLLT (p) group). SD: standard deviation; HILT: high-intensity laser therapy group; HILT (p): high-intensity laser therapy sham group; LLLT: low-level laser therapy group; LLLT (p): low-level laser therapy sham group.

Kruskal-Wallis analysis of variance (ANOVA) for independent variables did not show any differences between the studied groups. The LLLT and HILT laser procedures did not lead to a significant improvement in postural sway in patients with NSLP compared with standard stabilization training based on short- and long-term observations. Unfortunately, the applied irradiations appeared useless in all measures of postural stability parameters (Tables 2 to [Table t03] [Table t04] [Table t05] [Table t06] 7).

## Discussion

The Cochrane Back Review Group (22) assessed the effects of LLLT in patients with nonspecific NSLP. The authors searched CENTRAL Cochrane Library and common databases, such as MEDLINE, CINAHL, EMBASE, AMED, and PEDro, from their start to November 2007. Seven heterogeneous English language randomized controlled clinical trials (RCTs) were identified, and then studies were qualitatively and quantitatively analyzed according to Cochrane Back Review Group guidelines. It was concluded that there are insufficient data to draw firm conclusions on the clinical effects of LLLT for NSLP. The need for further methodologically rigorous RCTs assessing the effects of laser therapy for NSLP with comparisons to other therapies, different lengths, wavelengths, and dosages was highlighted. Therefore, we believe that our study meets this expectation.

There is only one meta-analysis of RCTs with blinded assessment of the outcome by Glazov et al. (23) that determines the effects of LLLT (including laser acupuncture) and its specific benefits in chronic NSLP. The authors established the following primary outcomes: pain measured by visual analog scale (VAS) or numerical pain rating scale (NPRS) measured immediately (<1-week posttreatment) and at short-term (1–12 weeks) follow-up as well as global assessment of improvement (dichotomous categorical outcomes of overall improvement or satisfaction with the received intervention). The secondary outcomes included range of back movement, adverse effects, and disability by the Oswestry Disability Index (ODI) or the Roland-Morris Disability Questionnaire (RMDQ) assessed at intermediate- (6 months) and long-term (1 year) follow-up. After selection of the records, 15 studies involving 1039 patients who satisfied the inclusion criteria were included. The authors demonstrated a moderate quality of evidence (GRADE) to support short-term clinical effectiveness of LLLT for chronic lumbar pain. It should be noted that higher laser dose procedures in patients with a shorter duration of back pain yielded the most significant clinical improvement.

The most recent single-blind RCT by Kolu et al. (24) compared the effects of HILT and a combination of transcutaneous nerve stimulation (TENS) with ultrasound therapy (UST) on pain intensity (VAS) and functional status (ODI) in 54 patients with lumbar pain caused by chronic radiculopathy. The patients were randomly divided into two groups: the first group (n=27; received 10 sessions of a combination of hot-pack, TENS, UST, and isometric lumbar exercises) and the second group (n=27; received hot-pack, isometric lumbar exercises, and HILT: 25 Hz, 10 W with 12 J/cm^2^). Immediately after the study, significant improvements for all measures were shown in both groups. However, it should be noted that in the four-week follow-up, statistically significant differences in VAS and ODI were noted for patients in group 1, indicating that TENS and UST combined with exercises were more effective than HILT combined with exercises.

Iranian scientists (1) in an RCT with concealed allocation, blinded assessors, and intention-to-treat analysis assessed whether six-week LLLT is an effective adjuvant treatment for chronic lumbar pain among 61 patients. The first group received laser therapy alone (810 nm, 27 J/cm^2^), the second received the same laser therapy and exercises, and the third group received placebo laser therapy and exercise. Laser therapy was performed twice a week for 6 weeks. No significant effect of laser therapy was noted compared with exercise for any outcome (Schober test, VAS, and ODI) at 6 or 12 weeks.

Gur et al. (2) performed an RCT that included 75 patients with lumbar pain divided into three groups: the first received LLLT (10.1 cm^2^ energy density, 2.1 kHz pulse frequency, 10 W diode power, 4.2 mW average power, 1 cm^2^ surface) on each painful point and stabilization exercises, the second received laser alone, and the third underwent exercise alone. VAS, Schober test, flexion and lateral flexion measures, RMDQ, and Modified Oswestry Disability Questionnaire (MODQ) were used in the clinical and functional evaluations pre- and post-interventions. Significant improvements regarding all outcome measures were noted with the exception of lateral flexion (P<0.05). Thus, the authors concluded that LLLT seemed to be an effective therapy in reducing pain and improving the functional ability for chronic lumbar pain.

A Brazilian RCT study (5) evaluated the effectiveness of LLT and LED radiation therapy associated with lateral decubitus position and exercises of the lower extremities in patients with lumbar hernia. A group of 54 subjects were assigned into groups: LLLT treatment using 904 nm (n=18), placebo LLLT (n=13), and LED treatment using 945 nm (n=18). Measurements included VAS, the degree of flexion of the affected hip with the universal goniometer, and functional capacity assessed with the ODI. An intergroup comparison showed a statistically significant improvement in VAS, hip mobility, and ODI in all groups P≤0.001). Statistically significant differences in radicular pain between the groups, gait claudication, and ODI were noted. However, a lack of differences was noted between the LLLT and placebo groups. Thus, it was concluded that the improvement in tested variables was associated with physical exercises performed as a basic therapy for all participants in this study.

In addition, a Polish study by Zdrodowska et al. (25) compared the effect of LLLT and pulsating magnetic field therapy (PMFT) on pain and range of motion of the spine among 120 adults suffering from degenerative spine disease and lumbar pain. Patients were divided into two groups: A (n=60; LLL: λ=820 nm, P=400 mW, Ed=6-12 J/cm^2^) and B (n=60; PMFT: 5 mT, 30 Hz, 15 min). The following assessments were used: VAS and the Modified Laitinen Questionnaire (MLQ) for pain intensity as well as the Schober test and the fingertip-to-floor test for spine mobility. The main findings indicated that both LLLT and PMFT decrease pain and increase spine mobility. However, LLLT showed a better analgesic effect, and PMFT presented greater spine mobility. A control group was lacking for this study; thus, it was not possible to make an unambiguous conclusion regarding which physical procedure was superior.

Koldaş Doğan et al. (3) conducted a double-blinded randomized clinical trial aimed at comparing the effectiveness of two different LLLT regimens on pain (VAS), lumbar range of motions (Schober test), and functional capacity (MODQ) in patients with chronic low back pain. The first group (n=20) received hot-pack as a warm-up combined with LLLT using a Ga-Al-As laser (λ=850 nm). The second group (n=29) received the same hot-pack combined with LLLT using a Helium-Neon laser (He-Ne, λ=650 nm) and Ga-Al-As combined plaque laser (λ=785/980 nm) for 15 sessions. Statistically significant improvements in all studied outcomes were noted for both LLLT methods. It was concluded that LLLT applied with combined wavelengths of He-Ne and Ga-Al-As shows a greater clinical effect; however, no superiority of the two different LLLT on pain level was detected.

Another Turkish RCT study by Boyraz et al. (12) assessed the effectiveness of HILT and UST in 65 patients diagnosed with lumbar disc herniation. All included patients were randomly divided into three groups: group 1 (n=20, 10 sessions of HILT, 1064 nm, 3.8 W, and 1800 J total dose), group 2 (n=25, 10 sessions of pulsed UST, 3 MHz, 50%, 1.5 W/cm for 6 min), and group 3 (n=20, pharmacotherapy for 10 days and isometric exercises of lumbar region). VAS scale, ODI, and SF-36 questionnaire for quality of life were used to measure clinical parameters, and 3-month follow-up data were provided. The researchers found that HILT and UST combined with exercise showed significant changes in most measured parameters among patients with lumbar pain. It should be pointed out that ten days following treatment, there was no significant difference between the groups compared with baseline values.

In their randomized, blinded placebo-controlled trial, Alayat et al. (6) assessed the effect of HILT alone or HILT combined with exercises in 72 male patients suffering from lumbar pain. Patients were randomly assigned into the following groups: first (HILT: 1064 nm, 50 J/cm^2^ plus exercises), second (placebo laser plus exercises), and third (HILT alone: HILT: 1064 nm, 50 J/cm^2^). The treatment program in all groups was continued for four weeks. Outcomes measured included a range of movement of the lumbar spine, pain level (VAS), and functional disability (RDQ and MODQ). There were no significant differences between the second (placebo) and third (HILT) groups, and the first group (HILT with exercises) provided no advantage compared with the other groups. It should be emphasized that a noticeable improvement in studied variables within groups was noted in the short-term analysis, and relapse occurred two months following the therapeutic interventions (similar to our study).

Another RCT study assessing short-term effects of HILT *vs* UST in the treatment of lumbar pain by Fiore et al. (13) was performed on 30 patients with no between-group differences at baseline in either VAS or ODI. Laser sessions were conducted using λ=1064 nm and E=1200 J of the total dosage (similar to our study). The experimental protocol described by the Italian researchers included 15 treatment sessions in 3 weeks and showed a significant reduction in pain (VAS) and a significant improvement in related disability (ODI) in patients from the HILT group compared with the UST group.

Choi et al. (4) studied the effectiveness of HILT in 20 patients with chronic lumbar pain who were assigned to two groups. Group 1 (n=10): patients received HILT and standard physiotherapy, including thermal compresses, UST, and electrotherapy. Group 2 (n=10): patients underwent only the above-mentioned conventional physical therapy (without HILT). All patients received therapy three times a week for four weeks. Measurement tools for pre- and post-assessment included VAS and ODI. After the end of the treatment sessions, both the VAS and ODI were significantly decreased in an intragroup comparison of the first group. In intergroup comparisons, HILT showed a significantly reduced VAS and ODI compared with the standard physiotherapy group.

Notarnicola et al. (14) randomized 66 patients with lumbar pain into three different LLLT protocols with constant parameters of P=5 W and E=50 J/cm^2^ for ten daily sessions. However, the protocols differed in terms to wavelengths: 650 nm (group 1), 810 nm (group 2), and simultaneous emission of 810 nm, 980 nm, and 1064 nm (group 3). VAS, ODI, and RMDQ were used as measurement tools before treatment (T0), at the end of the treatment sessions (T1), and at the 1-month (T1), 2-month (T2), and 4-month follow-up (T4). The authors showed that all wavelengths analyzed proved to be effective for lumbar pain. At T1 in all groups, a statistically significant improvement of all analyzed parameters was noted (P<0.01), which was maintained in long-term assessment (T4). Group 2 showed better remission in VAS and ODI at T4 (p=0.01). Comparing T0-T1, a significant improvement in RMDQ in patients treated with 810 nm (P<0.01) was noted, and this wavelength appeared to show the greatest promotion of neurodegeneration and modulation of nociception.

It is assumed that the basic mechanisms of laser therapy with different output powers include analgesic, restitution, and anti-inflammatory effects. These findings were mainly confirmed in animal experiments but not in clinical trials. There is an interesting report by Puhl et al. (26) who searched systematically for RCTs with placebo interventions for lumbar pain that used sham UST, sham laser, or sham drug therapy as the placebo control. They reported clinically meaningful alterations in pain findings following the use of sham oral medications for the treatment of NSLP.

So far, researchers have not analyzed the effects of laser therapy on postural control. However, in the literature there are many interesting cross-sectional studies comparing postural control between persons with chronic lumbar pain and the healthy population. Lafond et al. (27) recruited twelve adult subjects with chronic lumbar pain and 12 healthy controls without a history of musculoskeletal disorders. The inclusion criteria for study participation in the first group were chronic lumbar pain for at least 6 months, radiating pain no further than the buttocks, and normal neurological examination. Most subjects did not have a more specific diagnosis than mechanical lumbar pain. The exclusion criteria were a history of neurological disease or vestibular affliction, a history of dizziness, and medication with known effects on balance. The purpose of that study was to analyze the control of posture in subjects with chronic lumbar pain during prolonged standing. Ground reaction forces and moments were acquired from the force platform. Analogue signals were sampled at a frequency of 100 Hz and filtered with a zero-lag sixth-order Butterworth low-pass filter at 10-Hz cut-off frequency. COP displacements were computed in the anteroposterior (A-P) and medio-lateral (M-L) directions. Two different types of COP analysis were performed. First, structural analysis identified 3 COP postural patterns: a) shifting: fast displacement of the average COP position from one region to another (step-like); b) fidgeting: fast and large displacement, followed by a return of COP to approximately the same position (pulse-like); and c) drifting: slow, continuous displacement of the average COP position (ramp-like). The researchers also performed time and frequency domain analyses to obtain summary measures of COP signals in both the A-P and M-L directions: a) root mean square (RMS); b) mean COP speed; c) mean COP power frequency (COP frequency); and d) COP area. Lafond et al. (27) expected that during prolonged standing, postural control variables (COP patterns and postural sway) would show more deterioration in chronic lumbar pain subjects than in healthy subjects. Three main findings emerged from that investigation. First, results suggest that individuals with lumbar pain tend to exhibit less postural changes during prolonged standing than healthy adults, particularly in the A-P direction. Authors also found that during prolonged standing, chronic lumbar pain subjects swayed less than healthy adults. These two observations did not support the first hypothesis. The researchers expected greater postural changes in chronic lumbar pain subjects compared to healthy subjects during standing. However, the second hypothesis was confirmed. During quiet standing trials, prior to and after the prolonged standing period, chronic lumbar pain subjects presented greater postural sway than healthy subjects.

However, in contrast to the study above, the increased postural changes in patients with chronic lumbar pain in all directions during standing has been shown by other researchers (28). Further studies (especially based on a large population) are still needed.

### Novelty and limitations of the study

Our study is currently the only one of its type worldwide to evaluate the effectiveness of both HILT and LLLT with a homogeneous population of patients with chronic NSLP in the field of analysis of posture stability parameters. Of note, previous studies used only subjective measurement tools (questionnaires, surveys, scales) for assessment of clinical parameters. Novel elements also include the evaluation of early and follow-up findings and attempts to determine the placebo effect of laser therapy using a placebo-controlled study protocol. Based on an in-depth review of international medical databases, such as PubMed, MEDLINE, Scopus, EBSCOhost, PEDro, and Web of Science, it was not possible to identify a similar publication, which certainly confirms the innovative aspect of our efforts.

On the other hand, this fact makes it impossible to compare our results with those obtained by other researchers. This notion is all the more significant because the results obtained in our study were an unexpected disappointment because we wanted to show the clinical utility of laser irradiation in the area of measured indicators, which has not been confirmed. Although we used the technical parameters recommended in the literature and similar laser irradiation methodology to that reported in other papers, the obtained results were extremely surprising. Therefore, we strongly believe that the collected material should be verified by other researchers, which will allow for unambiguous verification of the results obtained in this study and facilitate access to evidence-based science.

Based on our experience, it can be concluded that there is a theoretical mechanism for reducing inflammation and eliminating pain in lumbosacral discopathy. Unfortunately, it does not correspond to functional improvement in the recovery of postural control and stability. Our team will certainly continue to research laser irradiation protocols because it is worth expanding the methodology to use adequate objective measurement tools (e.g., Biodex isokinetic system, surface electromyography device, or goniometry pendulum test) (24) and increase the sample size in specific groups. We did not provide the COP analysis for each leg separately. These facts certainly represent study limitations and should be improved in the future.

In conclusion, LLLT and HILT laser therapy did not lead to significant improvements in postural sway in patients with NSLP compared with standard stabilization training based on both short- and long-term observations. Further studies aimed at the objective evaluation of the effectiveness of laser irradiation should be performed. Moreover, further well-designed studies are needed to carefully verify the results of our study.
